# 分散固相萃取-高效液相色谱法测定水产品中7种麻醉剂

**DOI:** 10.3724/SP.J.1123.2021.08002

**Published:** 2022-02-08

**Authors:** Fang SHI, Dan SHOU, Micong JIN, Hongwei WANG, Xuguang CHEN, Yan ZHU

**Affiliations:** 1.浙江大学化学系, 浙江 杭州 310028; 1. Department of Chemistry, Zhejiang University, Hangzhou 310028, China; 2.浙江省中医药研究院中药研究中心, 浙江 杭州 310007; 2. Department of Medicine, Zhejiang Academy of Traditional Chinese Medicine, Hangzhou 310007, China; 3.宁波市疾病预防控制中心, 浙江 宁波 315010; 3. Ningbo Municipal Center for Disease Control and Prevention, Ningbo 315010, China; 4.浙江省立同德医院, 浙江 杭州 310012; 4. Tongde Hospital of Zhejiang Province, Hangzhou 310012, China; 5.浙江省冶金研究院有限公司, 浙江 杭州 310007; 5. Zhejiang Metallurgical Research Institute Co., LTD., Hangzhou 310007, China; 6.浙江省微量有毒化学物健康风险评估技术研究重点实验室, 浙江 杭州 310028; 6. Key Laboratory of Health Risk Appraisal for Trace Toxic Chemicals of Zhejiang Province, Zhejiang University, Hangzhou 310028, China

**Keywords:** 分散固相萃取, 高效液相色谱, 麻醉剂, 水产品, dispersive solid-phase extraction (DSPE), high-performance liquid chromatography (HPLC), anesthetic drugs, aquatic products

## Abstract

随着麻醉剂广泛用于渔业生产过程和水产品运输等领域,建立水产品中麻醉剂残留的检测方法具有重要意义。由于水产品基质复杂且麻醉剂残留水平低,因此需要合适的前处理方法以提高检测灵敏度。该研究基于分散固相萃取-高效液相色谱,建立了一种同时检测水产品中普鲁卡因、丁氧卡因、三卡因、丁香酚、甲基丁香酚、异丁香酚、甲基异丁香酚7种麻醉剂的分析方法。前处理采用分散固相萃取;确定了1.0%甲酸乙腈为提取溶剂,20 mg苯乙烯-甲基丙烯酸缩水甘油酯聚合物微球(PS-GMA)、50 mg *N*-丙基乙二胺(PSA)和10 mg C18混合吸附剂为净化剂,二甲基亚砜(DMSO)辅助氮吹的前处理方法;优化了提取时间和DMSO用量等条件。7种麻醉剂采用Welch welchrom C18色谱柱(250 mm×4.6 mm, 5 μm)进行分离,以甲醇和0.05%甲酸-5 mmol/L乙酸铵水溶液为流动相进行梯度洗脱,检测波长为235、260和290 nm,以鱼肉和对虾两种基质匹配标准曲线进行定量分析。实验结果表明,在优化的实验条件下,7种目标麻醉剂在各自的浓度范围内具有良好的线性关系(相关系数*R*^2^>0.999),检出限(LOD)为0.011~0.043 mg/kg。在鱼肉样品中,3个水平的平均加标回收率为79.7%~109%,相对标准偏差(RSD)低于7.2%;在对虾样品中,平均回收率为78.0%~99.9%, RSD低于8.3%。该方法具有快捷简便、操作简单、灵敏度高等优点,可应用于水产品中3种氨基苯甲酸酯类和4种丁香酚类麻醉剂的检测。

## Abstract (en)

Nowadays, anesthetics are widely used in fishery production processes, such as fish breeding, surgery, and fresh aquatic product transportation. Because of the widespread application of anesthetic drugs in aquatic products, there is an increasing demand for the rapid and sensitive detection of anesthetic drugs in aquatic products. The complex aquatic product matrix contains a variety of interfering substances, such as proteins, fats, and phospholipids, along with anesthetic drug residues at very low concentrations; therefore, it is necessary to adopt appropriate pretreatment methods for improving the sensitivity of detection. In this study, a dispersive solid-phase extraction (DSPE) method, combined with high-performance liquid chromatography, was established for the simultaneous detection of seven anesthetic drugs in aquatic products, viz. procaine, oxybuprocaine, tricaine, eugenol, methyl eugenol, isoeugenol, and methyl isoeugenol. For the DSPE step, pretreatment conditions, such as extraction solvent, extraction time, adsorbent amount, and DMSO dosage, were optimized. Sample pretreatment is a three-step process. First, in ultrasound-assisted extraction, 2.0 g samples were extracted using 10.0 mL 1.0% formic acid in acetonitrile under ultrasound conditions for 10 min. Then, DSPE was performed with mixed adsorbents: the solvent extracts were cleaned using 20 mg poly(styrene-glycidylmethacrylate) microspheres (PS-GMA), 50 mg primary secondary amines (PSA), and 10 mg C18, followed by separation by centrifugation. Finally, DMSO-assisted concentration was applied: the organic layer was collected and was dried at 40 ℃ in a N_2_ stream with 100 μL DMSO. Water was added to the residue to obtain a final volume of 1.0 mL for HPLC analysis. The seven anesthetic drugs were separated on a Welch welchrom C18 column (250 mm×4.6 mm, 5 μm) by gradient elution using methanol and 0.05% formic acid in 5 mmol/L ammonium acetate aqueous solution as mobile phases. The detection wavelengths were 235, 260, and 290 nm. Two matrix matching standard curves for fish and shrimp were applied for quantitative analysis. Under optimized conditions, the seven target anesthetics showed good linear relationships in their respective concentration ranges (*R*^2^>0.999), with the limit of detection (LOD) ranging from 0.011 to 0.043 mg/kg. In fish samples, the mean recoveries obtained at three concentration levels were between 79.7% and 109%, with relative standard deviations (RSDs) being less than 7.2%. In shrimp samples, mean recoveries were 78.0%-99.9%, with RSDs being less than 8.3%. This simple, rapid, accurate, and sensitive method can be applied to the detection of three kinds of aminobenzoic acid esters and four kinds of eugenol anesthetic drugs in aquatic products.

随着生活水平的提高,消费者对于水产品的质量和鲜活程度提出了更高的要求。麻醉剂是一种能在不同程度上抑制动物中枢神经系统功能的药物^[1]^。如今麻醉剂已经广泛用于鱼类的育种、手术等渔业生产过程和鲜活水产品运输等领域。在水产品运输过程中,麻醉剂的使用可以保持水产品的鲜活度,降低水产品的新陈代谢,从而减少因缺氧和皮肤损伤等造成的死亡,提高存活率^[2,3]^。

MS-222和丁香酚类麻醉剂是目前常用的渔用麻醉剂。MS-222又称三卡因,是美国、欧盟、加拿大允许使用的水产品麻醉剂,美国限定休药期为21天,最大残留限量(maximum residue limit, MRL)为1 mg/L;加拿大限定休药期为5天^[4]^。丁香酚是日本允许使用的鱼类麻醉剂,限定MRL值为0.05 mg/kg^[1]^;异丁香酚则在澳大利亚、新西兰等国家批准使用^[5]^,但我国并没有出台相关法规规范其使用和残留限量。虽然这些麻醉剂是安全的,但是也有报道提出一些麻醉剂具有致敏性和致癌性^[6]^,且美国国家毒理学计划(NTP)发布的数据显示,丁香酚类化合物对于啮齿动物是致癌物或者潜在致癌物,因此对水产品中麻醉剂残留的检测对于水产品的流通和质量管控等方面具有重要意义。

目前,用于水产品中麻醉剂的检测方法主要有高效液相色谱法(HPLC)^[7,8,9]^、液相色谱-质谱联用法(LC/MS)^[10,11]^、气相色谱-质谱联用法(GC/MS)^[12,13]^等。样品前处理是分析方法中的重要组成部分,由于水产品基质复杂,含有蛋白质、脂肪、磷脂等多种干扰物质,且水产品中麻醉剂残留的水平很低,因此需要通过适当的前处理方法提高检测的灵敏度。已经报道的相关前处理方法有固相萃取法(SPE)^[14,15]^、固相微萃取法(SPME)^[13,16]^、QuEChERS法^[17,18]^等。

分散固相萃取是在传统的固相萃取的基础上发展而来的一种前处理技术,通过将吸附剂分散于样品溶液达到富集和净化的效果,适用于液体样品和固体样品的液态提取物。固相萃取虽然具有重现性好、可实现自动化操作等优点,但是步骤复杂、成本较高^[19]^。分散固相萃取法则具有操作简单、有机溶剂使用量少、成本低、净化效率高等优点^[20,21]^,已经广泛应用于多种复杂样品基质中目标物的检测,如环境水样^[22,23,24]^、动物肌肉样品^[20,25]^、水果和果汁^[26]^、保健品片剂^[27]^等。本实验选取了常用的7种氨基苯甲酸酯类和丁香酚类麻醉剂作为分析物,采用1.0%甲酸乙腈为提取溶剂,苯乙烯-甲基丙烯酸缩水甘油酯聚合物微球(PS-GMA)、*N*-丙基乙二胺(PSA)和C18混合吸附剂为净化剂,结合高效液相色谱建立了水产品中普鲁卡因(procaine)、丁氧卡因(oxybuprocaine)、三卡因(tricaine)、丁香酚(eugenol)、异丁香酚(isoeugenol)、甲基丁香酚(methyl eugenol)、甲基异丁香酚(methyl isoeugenol)的检测方法。此方法操作简单、灵敏度与准确度高,可以为水产品中7种麻醉剂残留的检测提供参考。

## 1 实验部分

### 1.1 实验仪器与试剂

超高效液相色谱仪(Ultimate 3000,美国赛默飞公司),带有紫外检测器;KQ-500DE超声波清洗机(300 W,昆山市超声仪器有限公司); DCY-12S氮吹仪(绍兴市苏珀仪器有限公司); SU-70场发射扫描电镜(日本Hitachi公司); ASAP 2460比表面与孔隙度分析仪(美国Micromeritics公司)。

普鲁卡因盐酸盐、丁氧卡因盐酸盐、三卡因甲磺酸盐、丁香酚、甲基丁香酚、甲基异丁香酚、色谱纯的甲酸、乙酸铵均购自上海麦克林生化科技有限公司;异丁香酚、十二烷基硫酸钠(SDS)、苯乙烯(ST)、二乙烯基苯(DVB)、偶氮二异丁腈(AIBN)、聚乙烯吡咯烷酮(PVP)、甲基丙烯酸缩水甘油酯(GMA)、过氧化苯甲酰(BPO)、邻苯二甲酸二丁酯(DBP)、甲苯购自上海阿拉丁生化科技股份有限公司;苯乙烯、二乙烯基苯、偶氮二异丁腈、甲基丙烯酸缩水甘油酯、过氧化苯甲酰经纯化后使用;*N*-丙基乙二胺(PSA, 40~60 μm)、C18(40~60 μm)购买于上海安谱实验科技股份有限公司;苯乙烯-二乙烯基苯聚合物(PS-DVB,约5 μm)依照实验室已有技术制备而成^[28]^。色谱纯的甲醇、乙腈购自美国Tedia公司;分析纯的乙酸乙酯、无水乙醇购自国药集团上海化学试剂有限公司;所用超纯水均来自于Thermo Fisher Scientific超纯水系统。鱼和对虾样品购买于杭州本地超市。

### 1.2 实验方法

1.2.1 PS-GMA的合成

苯乙烯-甲基丙烯酸缩水甘油酯聚合物(PS-GMA)微球由实验室基于已有的成熟技术(两步溶胀法)^[29]^制备而成。合成步骤如下:将3 g聚乙烯吡咯烷酮和100 mL 95%乙醇加入三颈烧瓶并水浴加热至70 ℃。在N_2_保护下,在30 min内向其中逐滴加入0.8 g偶氮二异丁腈和18 g苯乙烯,250 r/min机械搅拌的条件下反应24 h。反应结束后用砂芯漏斗抽滤,并用去离子水洗涤干净。制备好的小球保存在10 g/L十二烷基硫酸钠水溶液中。将4.0 mL聚苯乙烯种子和20 mL 2 g/L十二烷基硫酸钠加入三颈烧瓶中,随后加入4.0 mL邻苯二甲酸二丁酯和30 mL 2 g/L十二烷基硫酸钠水溶液的混合乳化液,并在120 r/min机械搅拌的条件下反应24 h。向250 mL 10 g/L聚乙烯醇水溶液加入5 g甲基丙烯酸缩水甘油酯、10 g二乙烯基苯、14 g甲苯、0.2 g过氧化苯甲酰和0.8 g十二烷基硫酸钠,经过超声粉碎后加入三颈烧瓶对种子进行溶胀,反应24 h后通N_2_ 30 min,将水浴温度升高至70 ℃继续反应24 h。得到的小球抽滤后用热的去离子水和无水乙醇洗涤,随后以甲苯作为提取剂进行索氏提取48 h,产物用去离子水和无水乙醇洗涤至无甲苯味道,60 ℃真空干燥24 h,放于阴凉干燥处备用。

1.2.2 溶液的配制

准确称量普鲁卡因、丁氧卡因、三卡因、丁香酚、甲基丁香酚、异丁香酚、甲基异丁香酚标样,用甲醇/水(50∶50, v/v)配制成质量浓度为1000 mg/L的标准溶液,于-20 ℃冰箱中密封避光保存备用。

1.2.3 水产品前处理方法

鱼和对虾经清洗后吸干表面水分,鱼去鳞去皮,虾去头去壳,取鱼肉和虾肉分别于混合器研磨后置于-20 ℃冰箱中冷藏,实验前室温解冻备用。取2.0 g待测样品加入15 mL离心管中,加入10.0 mL 1.0%甲酸乙腈溶液,超声提取10 min, 8000 r/min下离心6 min,取上清液于另一15.0 mL离心管中,加入20 mg PS-GMA、50 mg PSA、10 mg C18,振荡2 min, 10000 r/min下离心6 min,取上清液加入100 μL二甲基亚砜(DMSO), 40 ℃下氮吹至近干,用去离子水定容至1.0 mL,过0.22 μm尼龙滤膜后进液相色谱分析。样品前处理大致流程如图1所示。

**图1:**
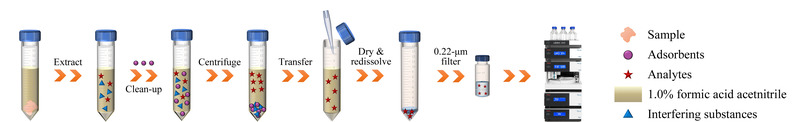
样品前处理流程示意图

1.2.4 色谱条件

色谱柱:Welch welchrom C18(250 mm×4.6 mm, 5 μm);柱温:30 ℃;进样体积:20 μL。流动相A:甲醇;B: 0.05%甲酸-5 mmol/L乙酸铵水溶液;流速:1.0 mL/min。梯度洗脱程序为:0~11 min, 20%A~80%A; 11~15 min, 80%A; 15~16 min, 80%A~20%A; 16~18 min, 20%A。检测波长:235、260和290 nm。

1.2.5 方法学验证

通过线性、检出限、定量限、精密度、准确度对该分析方法进行评估。基质加标标准曲线用于目标化合物定量分析;以信噪比的3倍和10倍计算检出限与定量限;相对标准偏差(relative standard deviation, RSD)用于评价日内与日间精密度,低、中、高3个水平(0.2、0.5、1.0 mg/kg)的加标样品用于评价方法的回收率。5个不同批次合成的PS-GMA用于评价方法的可重复性。

## 2 结果与讨论

### 2.1 PS-GMA的表征

采用扫描电镜对合成的PS-GMA微球进行表征,结果见图2。观察发现,合成的PS-GMA微球具有较好的球形,微球粒径约为13 μm,且具有良好的分散性。采用氮气吸附脱附法对PS-GMA微球的表面和孔的性质进行表征。根据Brunauer-Emmett-Teller (BET)等温线方程计算该微球的比表面积为333 m^2^/g,孔体积为0.36 cm^3^/g,平均孔径为4.35 nm。表征结果表明,合成的PS-GMA微球具有充足的空腔和比表面积,且分散性好,适合用于分散固相萃取吸附杂质以达到净化样品基质的目的。

**图2:**
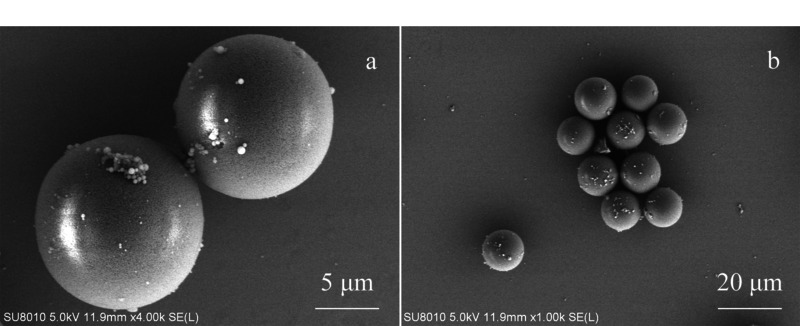
PS-GMA微球的SEM图

### 2.2 样品前处理条件的优化

2.2.1 提取溶剂的优化

样品的提取是复杂基质检测的重要步骤,提取溶剂的选择对提取效率有着重要的影响。本实验探究了乙腈、甲醇、乙醇、乙酸乙酯作为提取溶剂的提取效率,实验结果(见图3a)表明,4种提取溶剂对丁香酚类物质提取效果较好,但是对普鲁卡因、丁氧卡因、三卡因的提取效果较差。相比之下,乙腈的提取效果优于其他3种,由于乙腈对于鱼和对虾中的蛋白质具有一定的沉淀效果且能够减少脂肪的提取,因此选择乙腈进行后续实验。氨基苯甲酸酯类麻醉剂均为伯胺类化合物,向乙腈中添加甲酸有助于氨基的质子化,提高提取效率。对乙腈中甲酸的体积分数(0.1%、0.5%、1.0%、2.0%)进行了优化,结果如图3b所示。随着乙腈中甲酸添加量的增加,普鲁卡因、丁氧卡因、三卡因的回收率显著提升;当甲酸的体积分数达到1.0%时,各物质的回收率为80.3%~92.1%;继续增大甲酸浓度,回收率无明显提高。最后选择1.0%甲酸乙腈作为提取溶剂。

**图3:**
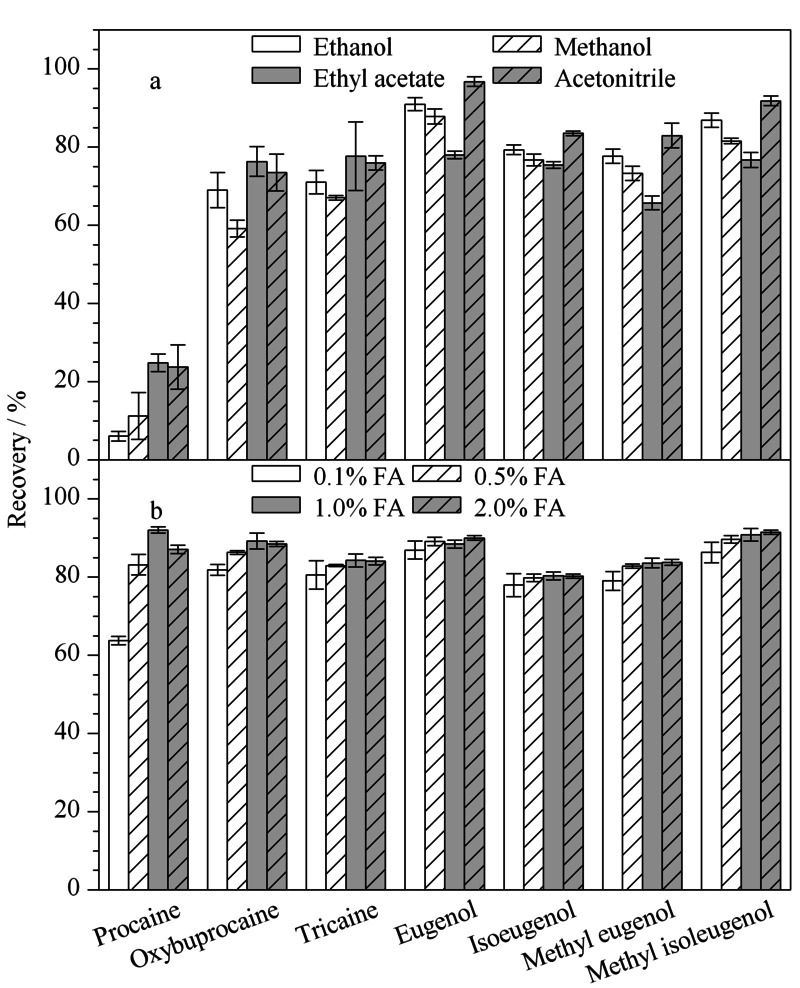
(a)提取溶剂种类和(b)乙腈中甲酸的体积分数对目标物回收率的影响(*n*=3)

2.2.2 超声提取时间的优化

超声有助于目标物更好地进行相转移,缩短提取时间,超声时间长短决定了目标物与提取溶剂的接触时间。本实验在0~20 min的范围内对超声时间进行优化,结果见图4。超声时间从0延长到10 min,各物质的回收率逐渐增加至80.0%~90.6%;但进一步延长超声时间,各物质回收率增长并不明显。综合以上实验结果,为节省时间和保证各物质的提取效率,选择10 min作为超声提取时间。

**图4:**
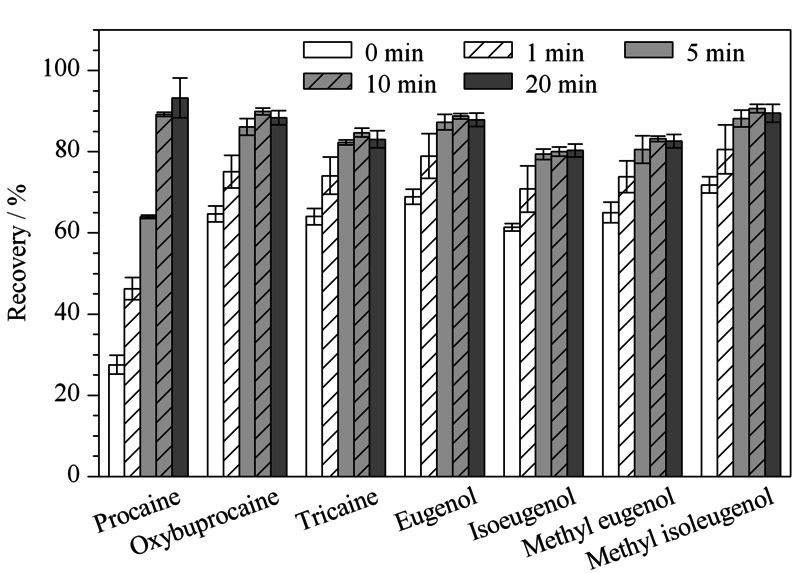
超声提取时间对目标物回收率的影响(*n*=3)

2.2.3 净化剂的优化

由于鱼肉和对虾样品基质复杂,共萃取物含有脂肪、蛋白质、色素等多种杂质,因此需要对样品基质进行净化。吸附剂的选择和使用量会影响分析的灵敏度和目标物的回收率。渔用麻醉剂种类和不同水产品基质均具有多样性,因此我们选择多种净化剂协同作用,以优化净化效果和净化流程,用于水产品中不同种类麻醉剂的检测。PSA作为一种弱的离子交换剂,可用于除去基质中的极性有机酸、脂肪酸、糖类等物质;C18可用于除去基质中的非极性化合物,如脂类等;PS-GMA这类聚合物微球具有大量苯环、大的比表面积,能够通过*π-π*作用和疏水作用去除基质中的脂肪、色素和非极性化合物。本实验对比了PS-DVB和PS-GMA两种聚合物微球的萃取效果,由图5a可知并无明显差别。鉴于PS-GMA表面存在环氧基,可通过开环反应对其进行修饰,方便在后续实验中进一步修饰、优化,所以本实验选用PS-GMA。保持50 mg PSA和10 mg C18不变,对PS-GMA的用量(0~40 mg)进行了考察。结果如图5b所示,当PS-GMA的用量增加到40 mg,各目标物的回收率有所降低,丁香酚类物质降低至64.7%~81.1%。这是由于吸附剂过量使用会造成对目标物的吸附,从而降低回收率。当PS-GMA的用量为20 mg时,各物质的回收率介于80.6%和96.6%之间。综合考虑净化效果和目标物回收率,最终PS-GMA的用量固定为20 mg。

**图5:**
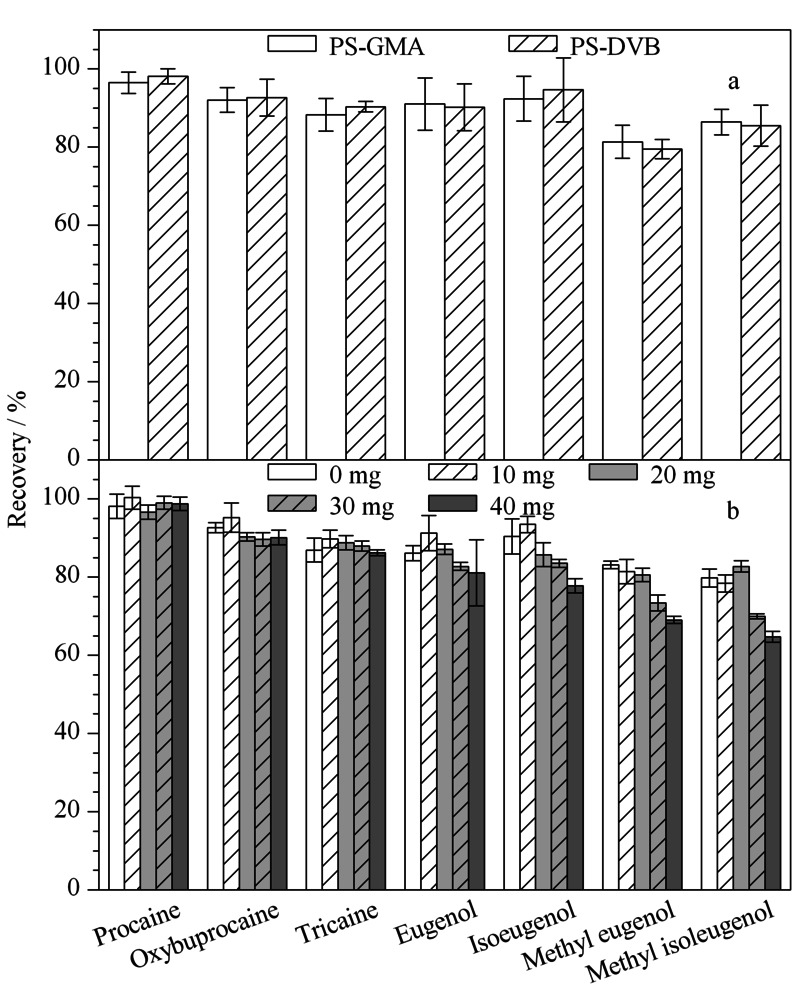
(a)吸附剂种类和(b)PS-GMA用量对目标物 回收率的影响(*n*=3)

2.2.4 DMSO用量的优化

样品经乙腈提取后,提取液体积大,且提取液与流动相存在较大差距,为避免溶剂效应的出现,样品氮吹浓缩后以去离子水定容。实验发现,提取后的加标样品直接在40 ℃氮吹后,普鲁卡因、丁氧卡因、三卡因3种物质的损失较小,而4种丁香酚类物质损失较大,回收率介于38.1%~52.2%之间。考虑到DMSO与水、乙腈等溶剂具有良好的相容性,本身沸点较高且对多种化合物具有良好的溶解性,尝试在氮吹前向提取液中加入少量DMSO,以减少丁香酚类化合物在氮吹过程中随溶剂蒸发造成的损失,提高回收率。本实验对DMSO的添加量进行了优化,结果见图6。随着DMSO加入量的增加(0~100 μL), 4种丁香酚类化合物的回收率明显提高;当DMSO添加量超过100 μL后,回收率无明显变化,因此选择添加100 μL DMSO再进行后续实验。

**图6:**
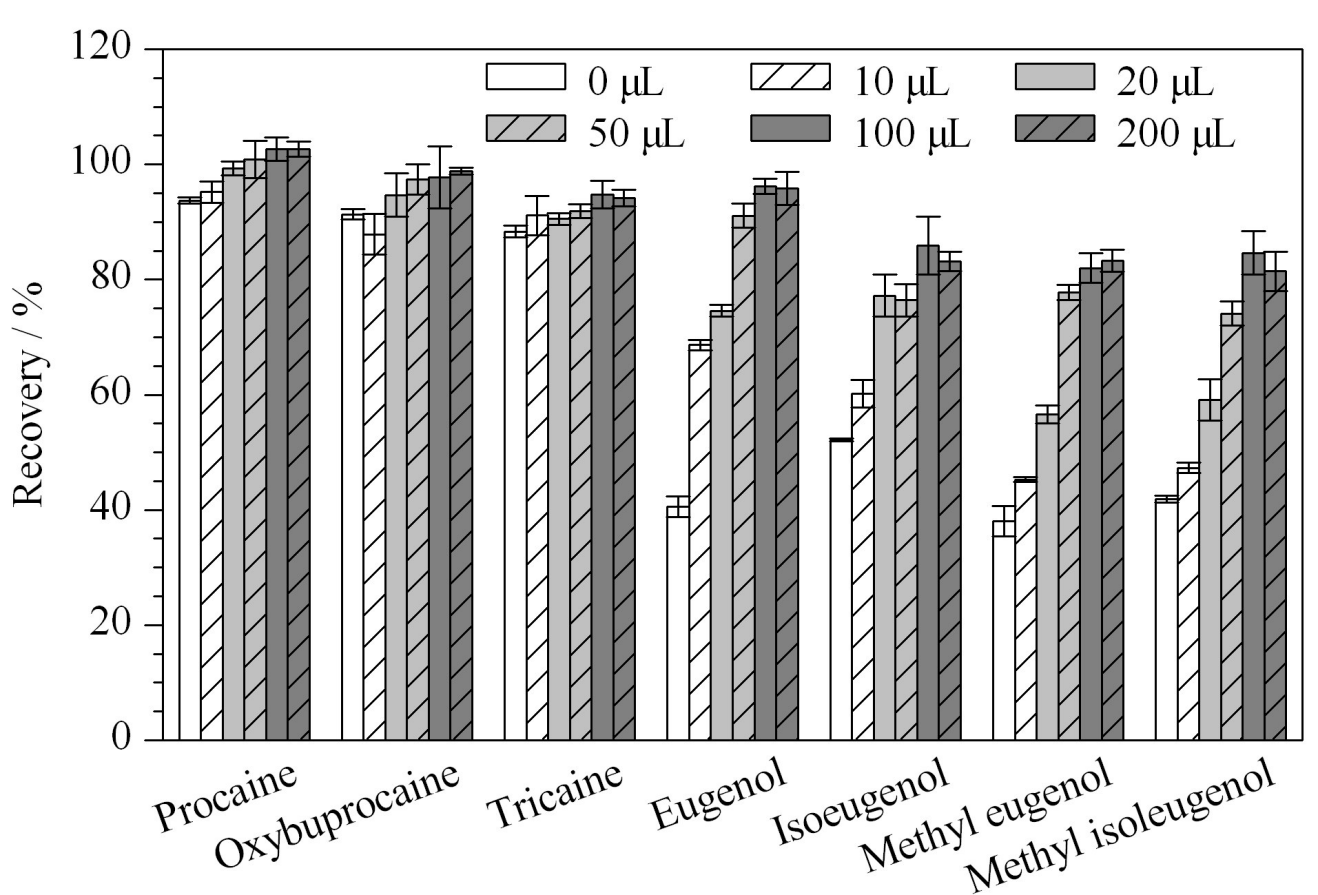
氮吹浓缩过程中DMSO添加量对目标物回收率的影响(*n*=3)

### 2.3 基质效应

实际样品基质复杂,而基质效应(ME)的存在可能会增强或者减弱目标物的响应信号,从而影响目标物的定性与定量,因此研究了本实验方法的基质效应。基质效应通过实际样品提取液为样品基质和以去离子水为样品基质所得的标准曲线的斜率之比进行衡量:

ME=(*K*_m_/*K*_s_-1)×100%

其中*K*_m_和*K*_s_分别指的是以鱼肉(或对虾)空白样品提取液为基质的标准曲线的斜率和以去离子水为基质的标准曲线的斜率。实验结果见表1,经过分散固相萃取净化后,鱼肉的基质效应介于-11.32%和0.23%之间,对虾的基质效应介于-9.32%到-0.70%之间,表明两种基质对于大部分目标物存在抑制情况。为保证实验的准确性,本实验通过基质匹配校准曲线进行定量分析。

**表 1:** 7种麻醉剂的线性方程、定量限、检出限和基质效应

Analyte	Linear range/(mg/L)	Matrix	Linear equation	R^2^	LOD/(mg/kg)	LOQ/(mg/kg)	ME/%
Procaine	0.05-10.0	fish	Y=0.9383X+0.0028	0.9999	0.011	0.036	-2.04
		shrimp	Y=0.9332X-0.0097	0.9997	0.011	0.037	-2.57
Oxybuprocaine	0.10-10.0	fish	Y=0.4977X-0.0106	0.9998	0.024	0.082	+0.24
		shrimp	Y=0.4930X-0.0249	0.9991	0.025	0.084	-0.70
Tricaine	0.10-10.0	fish	Y=0.5702X+0.0085	0.9999	0.023	0.077	-0.75
		shrimp	Y=0.5681X+0.0144	0.9992	0.024	0.080	-1.12
Eugenol	0.10-10.0	fish	Y=0.4011X-0.0052	0.9998	0.033	0.109	-3.02
		shrimp	Y=0.4036X-0.0253	0.9996	0.034	0.113	-2.42
Isoeugenol	0.05-10.0	fish	Y=1.0784X+0.0371	0.9995	0.013	0.044	-8.97
		shrimp	Y=1.1077X+0.0393	0.9994	0.013	0.043	-6.50
Methyl eugenol	0.20-10.0	fish	Y=0.4086X-0.0265	0.9998	0.043	0.142	-7.96
		shrimp	Y=0.4147X-0.0224	0.9997	0.043	0.144	-6.18
Methyl isoeugenol	0.10-10.0	fish	Y=0.9359X-0.0715	0.9993	0.021	0.070	-11.3
		shrimp	Y=0.9569X-0.0586	0.9992	0.021	0.071	-9.32

R^2^: correlation coefficient; Y: peak area; X: mass concentration, mg/L.

### 2.4 分析方法验证

2.4.1 线性范围、检出限和定量限

分别配制一系列鱼肉和对虾基质匹配标准溶液,按照1.2.3节条件进行液相色谱分析,得到7种麻醉剂的标样、鱼肉和对虾的加标(1.0 mg/kg)和未加标样品的色谱图(见图7)。以目标物的峰面积为纵坐标、质量浓度为横坐标,绘制基质匹配工作曲线,考察方法的线性范围、检出限和定量限,结果如表1所示。7种目标分析物在0.05~10.0 mg/L范围内具有良好的线性关系,相关系数*R*^2^均大于0.9991。分别以信噪比的3倍和10倍计算检出限和定量限,鱼肉样品中的检出限为0.011~0.043 mg/kg,定量限为0.036~0.142 mg/kg;对虾样品中的检出限为0.011~0.043 mg/kg,定量限为0.037~0.144 mg/kg。该方法的检出限均低于美国、日本、澳大利亚、欧盟等国家和地区对丁香酚类麻醉剂和MS-222在水产品中最高残留限定的检测要求。

**图 7:**
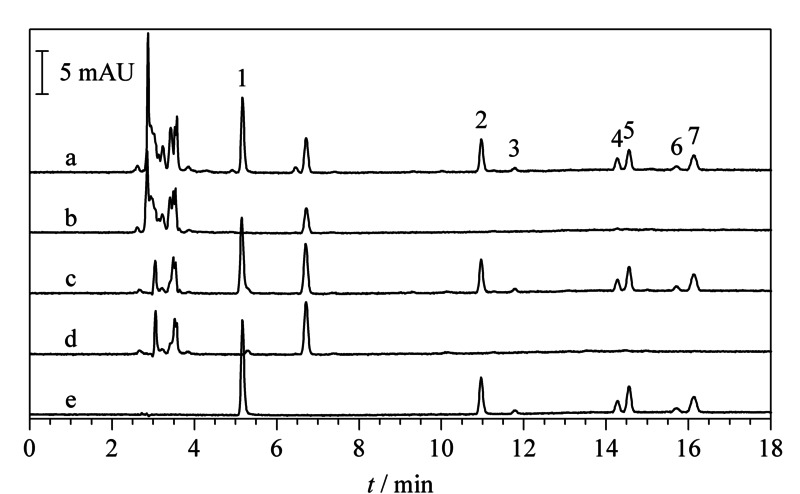
空白加标样品和7种麻醉剂混合标样的液相色谱图

如表2所示,比较本方法与已经报道的水产品中麻醉剂的检测方法^[8,9,30]^。本方法前处理过程分为8个步骤,耗时24 min,有机溶剂消耗量为10 mL,明显少于其他3种方法(液液萃取法^[8]^(12步,51 min, 26 mL)、冷冻干燥法^[9]^(18步,>63 min, 16 mL)、QuEChERS-SPE法^[30]^(19步,>31 min, 45 mL))。本方法可以简化前处理步骤、缩短前处理时间、减少有机试剂用量,是一种快速、简单、环保的前处理方法。

**表 2:** 本方法与已经报道的水产品中麻醉剂检测方法的比较

Sample	Analyte	Analytical method	Preparation method	Preparation time/min	Preparation steps	V(organic solvent)/mL	LOD/ (μg/kg)	Reference
Tilapia	eugenol	HPLC-UV	LLE	51	12	26	30	[8]
Ietalurus punetaus	eugenol	HPLC-UV	lyophilization	>63	18	16	45	[9]
Aquatic products	six anesthetics	HPLC-UV	QuEChERS-SPE	>31	19	45	60	[30]
Fish and shrimp	seven anesthetics	HPLC-UV	DSPE	24	8	10	11-43	this work

LLE: liquid-liquid extraction; DSPE: dispersive solid phase extraction.

2.4.2 方法的精密度与重复性

在空白鱼肉和对虾样品中添加混合标准溶液,在0.2、0.5和1.0 mg/kg 3个水平下进行加标回收试验,加标样品按照1.2.2节的方法进行前处理后液相色谱分析。一天内平行测定5次,计算日内精密度(intra-day RSD);平行测定5天,计算日间精密度(inter-day RSD),结果如表3所示。在鱼肉样品中,7种麻醉剂的平均回收率为79.7%~109%, RSD小于7.2%;在对虾样品中,7种麻醉剂的平均回收率为78.0%~99.9%, RSD小于8.3%;说明该方法具有良好的精密度。

**表 3:** 鱼肉和对虾空白样品在3个水平下的加标回收率和精密度(*n*=5)

Analyte	Spiked/ (mg/kg)	Fish				Shrimp			Batch-to-batch RSD/%
		Recovery/ %	Intra-day RSD/%	Inter-day RSD/%		Recovery/ %	Intra-day RSD/%	Inter-day RSD/%	
Procaine	0.2	79.7	6.2	7.2		82.0	3.1	5.5	6.3
	0.5	89.7	3.4	3.6		87.2	2.3	2.6	
	1.0	89.1	3.4	2.7		86.9	2.7	3.0	
Oxybuprocaine	0.2	89.8	2.8	2.3		93.9	2.2	6.0	2.1
	0.5	91.5	5.2	4.2		90.2	3.3	5.0	
	1.0	89.6	4.1	4.0		89.0	3.9	4.4	
Tricaine	0.2	83.5	5.1	2.6		78.0	5.5	2.7	5.0
	0.5	88.3	4.0	1.9		84.2	2.6	6.3	
	1.0	85.4	2.2	3.5		86.7	3.5	6.0	
Eugenol	0.2	91.2	6.8	2.7		83.2	3.3	5.0	6.7
	0.5	89.1	2.8	3.5		85.4	2.6	5.4	
	1.0	89.2	2.0	2.3		91.2	1.7	4.6	
Isoeugenol	0.2	84.7	4.2	7.2		94.8	3.2	8.2	4.3
	0.5	82.1	3.1	5.6		85.8	3.2	5.4	
	1.0	84.5	1.9	6.1		96.5	2.0	7.0	
Methyl eugenol	0.2	109.0	2.8	1.8		99.9	6.0	8.1	6.2
	0.5	101.0	4.6	3.8		98.0	2.5	3.8	
	1.0	94.1	2.3	3.8		95.0	1.3	5.1	
Methyl isoeugenol	0.2	106.0	4.1	3.3		93.0	6.7	3.1	5.3
	0.5	96.4	4.1	4.5		96.6	2.9	8.3	
	1.0	92.7	3.9	5.3		93.8	1.2	5.3	

采用5批不同时间制备的PS-GMA评价合成方法可重复性。实验结果表明,7种麻醉剂回收率的相对标准偏差小于6.7%,说明PS-GMA的合成具有良好的重复性。

### 2.5 实际样品的测定

采用已经建立的方法,对从当地市场随机购买的5份罗非鱼样品和5份对虾样品进行前处理和检测,10份样品均未检测出7种麻醉剂。虽然在实际样品中均未检测到7种麻醉剂,但实际样品加标回收试验可证明本方法的准确性和可靠性。

## 3 结论

本实验建立了一种同时检测水产品中3种氨基苯甲酸酯类和4种丁香酚类麻醉剂的DSPE-HPLC方法。样品通过1.0%甲酸乙腈结合超声提取,采用50 mg PSA+10 mg C18+20 mg PS-GMA净化后进样分析。样品前处理耗时24 min, 7种目标物在优化后的条件下具有良好的线性关系、回收率与精密度。该方法操作简单、耗时短、灵敏度高、有机试剂消耗少,可以应用于水产品中7种氨基苯甲酸酯类和丁香酚类麻醉剂的检测。本实验采用的PS-GMA表面的环氧基虽以原始状态存在,但其可进行开环反应,引入其他官能团,对PS-GMA进行功能化修饰,为提高吸附剂选择性提供可能。

## References

[1] Li J, Zhang J, Liu Y. Anal Bioanal Chem, 2015,407(21):6563 26092403 10.1007/s00216-015-8823-y

[2] Becker A G, Parodi T V, Heldwein C G, et al. Fish Physiol Biochem, 2012,38(3):789 21972065 10.1007/s10695-011-9562-4

[3] Wang W, Dong H, Sun Y, et al. J Appl Ichthyol, 2019,35(2):551

[4] Lü S W, Lei H T, Sun Y M. Food Science, 2012,33(1):267

[5] Zahl I H, Samuelsen O, Kiessling A. Fish Physiol Biochem, 2012,38(1):201 22160749 10.1007/s10695-011-9565-1

[6] Popovic N, Strunjak-Perovic I, Coz-Rakovac R, et al. J Appl Ichthyol, 2012,28(4):553

[7] Sun H, Lai J P, Chen F, et al. Anal Bioanal Chem, 2015,407(6):1745 25577355 10.1007/s00216-014-8420-5

[8] Huang W, Xu J L, Liu J F, et al. Journal of Food Safety and Quality, 2018,9(1):103

[9] Wang C X, Xiong G Q, Bai C, et al. Journal of Food Safety and Quality, 2019,10(8):2195

[10] Scherpenisse P, Bergwerff A A. Anal Chim Acta, 2007,586(1/2):407 17386741 10.1016/j.aca.2006.11.008

[11] Xie C, Li Q, Han G, et al. Biomed Chromatogr, 2019,33(5):e4512 30793335 10.1002/bmc.4512

[12] Li J, Liu H, Wang C, et al. Anal Bioanal Chem, 2016,408(24):6537 27531030 10.1007/s00216-016-9850-z

[13] Huang S, Xu J, Wu J, et al. Talanta, 2017,168:263 28391852 10.1016/j.talanta.2017.03.045

[14] Zheng X H, Sun T, Chen Y, et al. Food Science and Technology, 2020,45(4):333

[15] Ke C, Liu Q, Li L, et al. J Chromatogr B Analyt Technol Biomed Life Sci, 2016,1031:189 10.1016/j.jchromb.2016.07.048 27497157

[16] Botrel B M C, Abreu D C P, Bazana M J F, et al. Food Anal Methods, 2019,12(6):1390

[17] Li J, Liu H, Yu M, et al. Anal Methods, 2014,6(22):9124

[18] Xuan K Z, Liang Z G, Chen W H, et al. Physical Testing and Chemical Analysis Part B: Chemical Analysis, 2018,54(12):1405

[19] Tan X R, Zhao B, Lu J W, et al. Chinese Journal of Chromatography, 2022,40(1):57 34985216 10.3724/SP.J.1123.2021.03010 PMC9404128

[20] Sun P, Gao Y, Lian Y. Food Anal Methods, 2017,10(10):3217

[21] Aladaghlo Z, Fakhari A, Behbahani M. J Sep Sci, 2016,39(19):3798 27514993 10.1002/jssc.201600735

[22] Yu J, Di S, Ning T, et al. Microchim Acta, 2020,187(9):531 10.1007/s00604-020-04520-3 32862258

[23] Lou C, Wu C, Zhang K, et al. J Chromatogr A, 2018,1550:45 29615321 10.1016/j.chroma.2018.03.040

[24] Ji X F, Li S, Wu G G, et al. Chinese Journal of Chromatography, 2021,39(8):896 34212590 10.3724/SP.J.1123.2021.01006 PMC9404032

[25] Xu X, Feng T, Zhang J, et al. Anal Methods, 2018,10(42):5091

[26] Li S, Liang Q, Ahmed S A H, et al. Food Anal Methods, 2020,13(5):1111

[27] Cen J B, Liang Z S, Ou S J, et al. Chinese Journal of Chromatography, 2020,38(6):672 34213198 10.3724/SP.J.1123.2019.11008

[28] Zhong Y, Zhou W, Zhu H, et al. Anal Chim Acta, 2011,686(1/2):1 21237303 10.1016/j.aca.2010.10.041

[29] Guo D, Lou C, Wang N, et al. Talanta, 2017,168:188 28391841 10.1016/j.talanta.2017.03.053

[30] Gao P, Chen R M, Zeng D D, et al. Chinese Journal of Analysis Laboratory, 2018,37(1):88

